# The Relationships between Weather-Related Factors and Daily Outdoor Physical Activity Counts on an Urban Greenway

**DOI:** 10.3390/ijerph8020579

**Published:** 2011-02-23

**Authors:** Dana Wolff, Eugene C. Fitzhugh

**Affiliations:** Department of Kinesiology, Recreation, & Sport Studies, The University of Tennessee, 1914 Andy Holt Ave., HPER 303, Knoxville, TN 37996, USA; E-Mail: fitzhugh@utk.edu

**Keywords:** physical activity, weather, greenway, trail counts, environmental barriers

## Abstract

The purpose of this study was to examine relationships between weather and outdoor physical activity (PA). An online weather source was used to obtain daily max temperature [DMT], precipitation, and wind speed. An infra-red trail counter provided data on daily trail use along a greenway, over a 2-year period. Multiple regression analysis was used to examine associations between PA and weather, while controlling for day of the week and month of the year. The overall regression model explained 77.0% of the variance in daily PA (p < 0.001). DMT (*b* = 10.5), max temp-squared (*b* = −4.0), precipitation (*b* = −70.0), and max wind speed (*b* = 1.9) contributed significantly. *Conclusion:* Aggregated daily data can detect relationships between weather and outdoor PA.

## 1. Introduction

A greenway is commonly defined as a “linear open space established either along a natural corridor such as a riverfront, stream valley, or ridgeline, or overland along a railroad right-of-way converted to recreational use, a canal, a scenic road, or other route” [1]. Recently, the building and use of greenways has become popular both nationally and internationally [2]. The construction of greenways provides communities with a venue for outdoor physical activity (PA), especially walking and cycling, the two most common modes of leisure time PA [3,4]. Greenways developed in urban areas may be even more important due to the proximity to large population centers. Research does indicate that an individual is twice as likely to engage in outdoor PA if he or she perceives they have access to outdoor PA [5]. Therefore, it is important to understand the factors responsible for seasonal variation in outdoor PA as it may aid in the design and promotion of outdoor PA opportunities.

However, despite easy access provided by greenways and other locations (e.g., parks school playgrounds, *etc.*) for outdoor PA, individuals have a variety of potential barriers to being physically active in the outdoor environment. One of the barriers to overcome is weather, including both hot and cold temperature extremes, precipitation, wind, and humidity. Merrill and colleagues recently found that inclement weather is associated with lower rates of PA [6]. Climate is also related to variations in total PA with geographic areas of moist tropical conditions having a lower percentage of the population reporting PA levels meeting recommendations [7]. Seasonal trends have also been demonstrated in these studies indicating that PA is lower in colder months compared to warmer months [8–12].

Although the specific weather related factors influencing physical activity varies between studies [8,9], several climate related conditions have been found to be associated with outdoor PA including: precipitation [13,14], humidity [15], and temperature [8,12,14]. A study by Lindsey and colleagues [8] investigated weather and time-related variables to determine their correlation to neighborhood trail use. Results from this analysis indicate that temperature, precipitation, and snow fall all impact neighborhood trail use. Specifically, trail traffic increased 3.2 percent for every one degree Fahrenheit increase in temperature above the long-term mean and decreased by 40% for every inch of rain above the long-term mean. Month of the year and day of the week were also related to trail use, with weekend trail use being significantly higher than the weekday and trail use varying across months. While this study describes a relationship between weather and outdoor PA, this was not the main focus of the investigation. Thus, the investigators only measured three weather-related factors in this study, potentially limiting the ability to detect the true effect of weather on trail use. Instead of using daily weather measurements, long-term averages for temperature, precipitation, and snowfall were created from data provided by the National Oceanic and Atmospheric Administration. These long-term means were then used as the reference point for the change in each weather-related variable.

The aforementioned studies found that a relationship exists between various weather variables and PA, but most of these studies do not directly measure outdoor PA and weather. Instead, most of these studies use methodologies that utilize self-reporting methods such as focus groups, interviews, and telephone surveys. The Behavioral Risk Factor Surveillance Survey is a commonly used instrument which indirectly measures PA through self report and uses geographic location to determine weather conditions at participant’s location at the time of the interview [6]. This method, while insightful, is not specific to outdoor PA, is subject to recall bias, and may be inaccurate in matching of participant’s geographic location with weather conditions [7].

While the relationships between weather variables and outdoor PA have previously been examined, there is limited research directly measuring both outdoor PA and weather conditions simultaneously over several cycles of seasonal change. In addition, research has not focused on outdoor PA on an urban greenway. In light of these findings, the purpose of this study was to investigate the relationship between weather related factors and daily PA trail counts on an urban greenway.

## 2. Methods

This study examined the relationship between outdoor PA and weather, and was conducted from July 1, 2007 to August 2, 2009 and included a total of 611 days of valid PA trail counts and weather measures. Over the course of the study over 100 days of PA trail counts were lost due to environmental conditions (*i.e.*, insect infestation of trail counter) and equipment failure (*i.e.,* persons tampering with or batteries dying in trail counter).

### 2.1. Physical Activity Measurement

**Figure 1.**
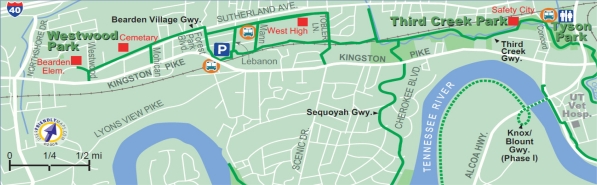
Third Creek Greenway (reproduced with permission from [17], © UserFriendlyMaps.com).

Outdoor physical activity, the dependent measure in this study, was measured as aggregated daily counts captured by an infrared trial counter located on a heavily used urban greenway [16]. The outdoor site for this study was on the mid-point of the Third Creek Greenway, an urban greenway centrally located in Knoxville, Tennessee, approximately two miles from the University of Tennessee campus [17]. The greenway is 4.5 miles in length and is easily accessible, connecting to three other greenways in the Knoxville area (Figure 1). Third Creek greenway is located in a mixed land use (residential and commercial) area and is surrounded by vegetation and moderately dense tree coverage most of the greenway [13].

An infrared trail counter (Ivan Technologies, Canton, CT), placed one meter above the ground, was used to measure daily trail PA counts (e.g., walking, running, cycling) on the greenway. The trail counter works by emitting an invisible infrared beam across the trail. When the infrared beam is broken the result is a recorded trail count with a time-stamp. Day and time counts from the infrared trail counter were downloaded in the field using the HOBO Shuttle which was then transferred to a laptop using BoxCar Pro 4.3 software (Onset Computer Corp., Bourne, MA). Downloads were performed once a month in low usage months and twice a month during months with heavy trail usage. Aggregated PA trail counts were measured by summing the total number of time-stamp counts for each 24-hour period.

Validation of the trail counters was previously conducted in a pilot study [16] to ensure that PA occurring at high speeds (e.g., cycling) was captured by the trail counter. Ten passing trials of a bike were conducted at speeds of 5, 10, and 15 mph. The trail counter accurately recorded all 30 trials (100%).

Additional validation of the trail counter was conducted on two separate occasions totaling four 2-hour periods of direct PA observation. During the direct observations, a total of 219 users were observed with 95% of users being counted by the trail counter during each observation session. Miscounts resulted from side-by-side users (*i.e.*, 2 or more trail users walking, jogging, or cycling past the trail counter at the same time), resulting in the infrared beam only being broken once. Walkers, joggers, and cyclists passing in a single file manner were captured by the trail counter regardless of the activity.

### 2.2. Weather Related Measures

Daily weather related measures were captured from Weather Underground, an online weather source [18]. Previous research indicates that public domain weather sources provide weather data that is just as valid and reliable as data obtained from an onsite weather station [16]. Weather factors utilized in this study included daily temperature (°F), dew point (°F), humidity (%), atmospheric pressure (in. Hg), wind speed (mph), and precipitation (in.). Each factor was measured using the maximum (max) and range for each day. The range was calculated using the high and low of each day and was used as an indicator of the weather changes that occurred throughout the day. Max temperature squared was calculated to detect the max temperature at which PA trail counts peak and begin to decline with increasing temperature

### 2.3. Statistical Analysis

In order to address the research questions of this study, several statistical approaches were utilized. Initially, descriptive statistics and correlations were conducted in order to determine normality of the data and multi co-linearity between weather-related measures. Weather-related measures found to be correlated were reviewed to determine which variables had the greatest correlation with mean PA trail counts. Based on this analysis it was determined that the following variables be used in step-wise multiple regression: max temperature, max temperature squared, max humidity, humidity range, dew point range, max wind speed, precipitation, year, and day of the week. Once regression analysis was performed, T-tests and ANOVAs were conducted to determine significant differences in mean PA trail counts between variables included in the regression model.

Statistical analyses were conducted using SPSS 17.0 software (SPSS for Windows 17.0, SPSS, Inc). Significance was set at p ≤ 0.05.

## 3. Results

**Figure 2.**
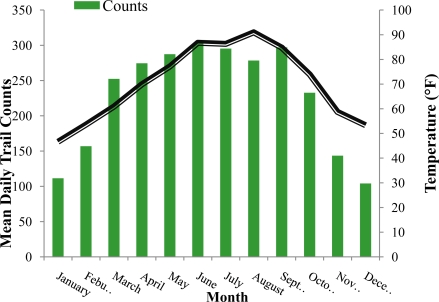
Relationship between mean monthly PA trail counts & mean monthly max temperature (July 2007–August 2009).

Figure 2 and Table 1 display trends in PA trail counts collected throughout the study period. PA trail counts were lower in winter compared to summer (Figure 2) with December having the lowest mean counts (104.1 ± 49.0) and June the highest (302.7 ± 86.0) (Table 1). Day of the week related to PA trail counts, with weekend PA trail counts being higher than weekday levels (p = 0.001) (Table 1). Table 2 reflects monthly changes in daily max temperature throughout the year with the lowest temperature seen in January (46.6 °F ± 10.7) and the highest in August (91.0 °F ± 5.8).

**Figure 3.**
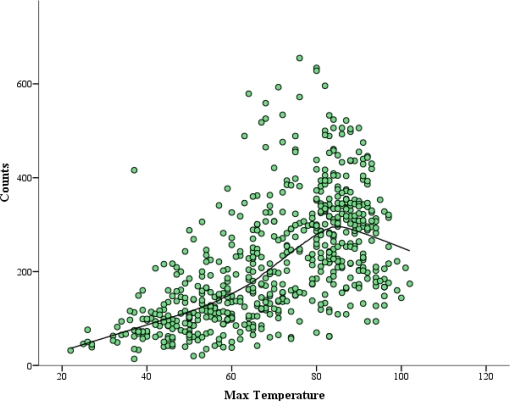
Relationship of daily max temperature with outdoor PA trail counts.

The association between outdoor physical activity and weather related factors measured by step-wise multiple regression found that weather measures and time-related factors (year and day of the week) explained 77.0 % of the variance in daily PA (Table 3).Max temperature had the greatest influence on daily PA with trail counts increasing by 10.5 for every 1 °F in temperature until peaking at 84 °F (Figure 3). For every 1 °F increase in temperature above 84 °F, trail counts decreased by 4 as reflected by max temperature squared (Figure 3). For every inch of precipitation trail counts decreased by 70 and for every 1 mph increase in max wind speed trail counts decreased by 1.92 (Table 3).

## 4. Discussion

The results of this investigation suggest that weather related factors are associated with changes in outdoor PA trail counts measured on an urban greenway. These results support our hypothesis that the relationship between weather and physical activity trail counts can be detected directly at the aggregated daily level. In comparison to previous research investigating this relationship, our study is one of few that objectively measures outdoor PA across multiple seasons [8,12,16]. Furthermore, the variance in PA trail counts explained by weather related factors in this study is at a level more than twice the amount reported in previous investigations [8,16,19,20]. A possible explanation for this finding resides with the 24-hour collection of PA trail counts. Previous research from Burchfield used hourly data during daylight hours which may have excluded greenway users during the early morning or late evening hours [16]. Therefore, greater variability and range in weather may better reflect actual weather for that particular day in addition to a better measure of actual trail use.

Consistent with previous research findings, results from this study indicates a curvilinear relationship between PA and weather exists (Figure 3) [16,20]. Results show a trend in PA trail counts with counts increasing in warmer and decreasing in colder temperatures (Figure 2), reflecting seasonal changes in PA (Table 2). The seasonal changes reflected in the data are similar to previous research by Hamilton *et al.* [9] and Duncan *et al.* [11] which found that both adults and children, respectively, increased their step counts in summer months and decreased in winter months. When controlling for day of the week and month, results for max temperature squared indicated that PA trail counts peaked at 84 °F and began to decrease at a rate of 0.045 counts for every 1 °F increase in temperature above 84 °F. Results for max temperature squared support findings by Burchfield *et al.* who found that at 75.8 °F, PA begins to decrease after controlling for all monthly and weekly factors and air quality [16].

When controlling for day of the week and month, we found that PA trail counts peaked at 84 °F and began to decrease at a rate of 0.21 counts for every 1 °F increase in temperature above 84 °F. This relationship is consistent with findings from Burchfield *et al.* [16] and Brandon *et al.* [14] who found that at 75.8 °F and 68 °F respectively, PA begins to decrease after controlling for monthly and weekly time-related factors [16]. It is our opinion that the difference in peak temperature is due to the difference in study design as both Burchfield *et al.* [16] and Brandon *et al.* [14] measured PA and weather on an hourly basis while this study utilizes daily measurements. Furthermore, weather and PA measures were only collected during daylight hours in Burchfield and Brandon’s studies whereas the measures in this study are based on data collected over a 24-hour period.

Precipitation and max wind speed were also significantly related to PA trail counts. Specifically, for each inch of accumulated precipitation during the day, outdoor PA decreased by 70.0 trail counts per day (Table 3). Decreases in outdoor PA were also related to increases in max wind speed with 1.9 fewer trail counts per day occurring for every 1 mph increase in max wind speed. While previous research has found precipitation results in decreases in PA [8–10,20]; this current study is only one of few studies demonstrating a significant relationship between wind and PA [12].

These results indicate that weather related factors account for a considerable amount of the variation in daily PA trail counts when measured on a daily basis. When compared to data collected on an hourly basis, daily measures explain more variance in the association between weather and physical activity (42% and 77%, respectively). Therefore, we believe aggregated daily data can be used to detect relationships between outdoor PA and the weather. These findings are important for researchers and urban planners when studying PA on trails and greenways, as this less burdensome level of analysis can be used without compromising precision. Future research is necessary to better understand health related behaviors and motivations of trail users in addition to how weather impacts indoor physical activity levels.

The present study has several strengths inherent in its design. First, the time period for this study ranged over three consecutive years, from July 2007 to August 2009. As well, our study is one of few studies assessing the relationship of daily aggregated weather-related factors with outdoor PA in a free-living environment. The objective measurement of PA in this study using an infrared trail counter also provides a more precise measurement of outdoor PA on an urban greenway.

There are also limitations that should be considered when interpreting the aforementioned results. First, outdoor PA was assessed with an infrared trail counter, which only provides an estimation of the number of individuals using the greenway. The possible inaccuracy of this estimation has been demonstrated by Lindsey and colleagues who found approximately 90% of trail users begin and end their trail use at the same location [8]. Results from our direct observation at the Third Creek Greenway found an underestimation of trail users occurred with approximately 13% of users passing the infrared beam simultaneously, thus only breaking the beam once. Results from the direct observation indicate there is a potential error rate of 5% of the infrared trail counter when estimating the number of greenway users.

Another limitation of this study is the location of the trail counters in the natural environment. Although the counter was placed in a discrete place along the trail, it can still be found and thus tampered with by the general public. Specifically during our study, data were lost due to the trail counter being turned off. Days were also lost due to invalid counts caused by the formation of a wasp nest in the lens of the trail counter, resulting in hundreds of extra counts per day. Over the course of the study these problems resulted in more than 100 days of lost PA data.

The lack of trail user characteristics also limits the inferences that can be made related to the influence of weather related factors on total PA. While results from this and other studies [8,14,16,19,20] indicate that temperature and outdoor PA are inversely related, a decrease in outdoor PA may not truly reflect decrease in total PA. Due to the lack of trail user information in this study it is possible many users may have substituted indoor PA for outdoor PA when weather was perceived to be a barrier to outdoor PA.

While the generalizability of our results is limited to geographic locations similar to the East Tennessee region, we believe research exploring variations in PA levels across different geographic locations is important in understanding and designing opportunities for outdoor PA. Based on our study, and the work of others, we also believe that effects of weather related factors should be considered when investigating changes in PA levels in outdoor environments.

Although the construction and use of urban greenways in the promotion of physical activity (PA) has become more predominant in our society, the environmental determinants (*i.e.*, weather, climate, *etc.*) impacting greenway usage are not well understood [2]. This is important to urban planners and public health officials, as this relationship has previously not been clearly defined. In terms of significance, information obtained related to the environment and outdoor physical activity are important in effectively developing and designing outdoor recreation facilities [2]. For example, trees could be planted along the greenway to shield against the elements, specifically, high wind and temperatures.

These results indicate that weather related factors account for a considerable amount of the variation in outdoor PA trail counts when measured on a daily basis. When compared to data collected on an hourly basis, daily measures explain more variance in the association between weather and physical activity (42% and 77%, respectively). Therefore, we believe aggregated daily data can be used to detect relationships between outdoor PA and the weather. Further research is necessary to better understand health related behaviors and motivations of trail users, in addition to how weather impacts levels of indoor physical activity.

These findings are important for researchers and urban planners when studying PA on trails and greenways, as this less burdensome level of analysis can be used when designing programs to promote outdoor PA. Additionally, future research investigating the relationship between weather and outdoor PA can assess these measures at a daily level. In areas that have unfavorable weather conditions for a large part of the year, understanding this relationship might be important for increasing outdoor PA. This might be done by incorporating buffers and shading such as trees throughout the greenway to shield against the elements, specifically, high wind and temperatures.

## Figures and Tables

**Table 1.** Mean daily PA Counts by year, month & day of the week (July 2007–August 2009).

**Year**	**N**	**Mean**	**SD**
2007	145	182.6	94.8
2008	284	219.1	118.5
2009	184	253.8	151.6
**Month**			
January	61	111.5	64.7
February	57	156.8	111.5
March	26	252.3	152.8
April	43	274.6	125.5
May	57	287.4	131.6
June	26	302.7	86
July	92	295.3	99.1
August	60	278.3	106.2
September	29	297.1	123.8
October	42	232.7	106.1
November	58	143.4	75.4
December	62	104.1	49
**Day of Week**			
Monday	86	195.1	109.2
Tuesday	87	194.7	113.6
Wednesday	87	201.2	118.9
Thursday	88	177.5	100.8
Friday	88	189.5	97.6
Saturday	89	304.3	129.6
Sunday	88	281.6	151.7

* N, total valid days; SD, standard deviation;

* Year: months represented for each year 2007 (Jul.–Dec.), 2008 (Jan.–Dec.), 2009 (Jan.–Aug.).

**Table 2.** Mean third creek greenway monthly weather conditions (July 2007–August 2009).

	**Max Temp**		**Temp Range**		**Max Dew Point**		**Dew Range**		**Max Humidity**		**Humidity Range**		**Max Atmospheric Pressure**		**Max Wind Speed**		**Precip.**	

**Month**	*x̄*	*(SD)*	*x̄*	*(SD)*	*x̄*	*(SD)*	*x̄*	*(SD)*	*x̄*	*(SD)*	*x̄*	*(SD)*	*x̄*	*(SD)*	*x̄*	*(SD)*	*x̄*	*(SD)*
**January**	46.6	(10.8)	19.6	(7.0)	34.2	(14.4)	15.0	(7.9)	86.9	(11.8)	38.8	(15.0)	30.3	(0.2)	17.6	(8.5)	0.2	(0.4)
**February**	53.6	(10.5)	21.5	(7.3)	37.8	(11.8)	14.7	(7.7)	84.0	(12.4)	39.7	(12.3)	30.2	(0.2)	19.4	(8.5)	0.1	(0.3)
**March**	60.9	(10.37)	21.2	(8.9)	43.5	(11.3)	12.4	(6.7)	85.7	(11.6)	41.9	(15.5)	30.2	(0.2)	18.5	(6.7)	0.1	(0.3)
**April**	69.9	(9.88)	22.2	(8.6)	50.6	(8.2)	11.5	(5.9)	86.9	(8.0)	46.2	(13.9)	30.1	(0.1)	18.7	(7.0)	0.1	(0.2)
**May**	77.2	(5.64)	20.4	(6.6)	60.2	(6.7)	9.9	(4.7)	90.2	(8.7)	43.4	(13.0)	30.0	(0.1)	19.0	(7.7)	0.2	(0.3)
**June**	86.7	(4.52)	20.8	(4.5)	67.3	(4.8)	7.8	(3.6)	90.7	(7.2)	45.3	(11.1)	30.0	(0.1)	18.2	(7.5)	0.1	(0.3)
**July**	86.3	(4.03)	19.5	(4.3)	67.3	(4.5)	7.2	(2.9)	90.4	(7.1)	43.8	(10.3)	30.1	(0.1)	16.8	(5.6)	0.2	(0.4)
**August**	91.0	(5.84)	21.9	(5.3)	67.9	(4.3)	8.2	(3.6)	85.8	(7.9)	46.7	(10.9)	30.0	(0.1)	14.0	(5.1)	0.1	(0.4)
**September**	84.8	(5.93)	22.0	(5.9)	63.6	(5.7)	8.6	(5.5)	88.7	(6.5)	48.7	(11.0)	30.1	(0.1)	14.0	(4.5)	0.1	(0.3)
**October**	74.0	(9.43)	23.8	(7.2)	53.1	(10.0)	10.2	(6.1)	88.1	(8.8)	49.4	(13.5)	30.2	(0.2)	13.8	(4.8)	0.0	(0.2)
**November**	58.7	(9.89)	22.3	(7.4)	42.0	(11.2)	12.5	(7.2)	87.1	(10.9)	44.5	(14.2)	30.2	(0.2)	15.7	(5.8)	0.1	(0.3)
**December**	53.4	(10.36)	19.1	(7.6)	43.0	(12.8)	14.1	(9.0)	92.0	(9.6)	35.7	(14.7)	30.3	(0.1)	16.9	(7.5)	0.2	(0.4)

x̄, mean; (SD), Standard Deviation; Temp (°F), temperature; Temp Range (°F), daily high temperature minus low temperature; Max Dew Point (°F); Dew Range (°F), daily high dew point minus low dew point; Max Humidity (%); Humidity Range (%), daily high humidity minus low humidity; Max Atmospheric Pressure (in. Hg); Max Wind Speed (mph); Precip. (in,), precipitation.

**Table 3.** Relationship between outdoor PA trail counts and weather-related factors (step-wise multiple regression).

**Variable**	**Unstandardized Coefficients**		**Standardized Coefficient**	**95% CI**	
	***b***	**Std. Error**	**Beta**		
Constant	−407.22	47.192	------	−499.899	−314.54
Max Temp	10.489	1.473	1.446	7.595	13.382
Year	57.608	4.639	0.331	48.497	66.72
Precipitation	−70.029	7.624	−0.259	−85.001	−55.057
Day of the Week	15.573	1.643	0.245	12.346	18.8
Max Temp Sq	−0.045	0.011	−0.823	−0.066	−0.023
Max Wind Speed	−1.920	0.514	−0.106	−2.930	−0.911

R^2^ = 0.77; p ≤ 0.001.
